# *In-vivo* oxidized albumin– a pro-inflammatory agent in hypoalbuminemia

**DOI:** 10.1371/journal.pone.0177799

**Published:** 2017-05-24

**Authors:** Faiga Magzal, Shifra Sela, Andrea Szuchman-Sapir, Snait Tamir, Regina Michelis, Batya Kristal

**Affiliations:** 1 Eliachar Research Laboratory, Galilee Medical Center, Nahariya, Israel; 2 Faculty of Medicine in the Galilee, Bar Ilan University, Safed, Israel; 3 Laboratory of Human Health and Nutrition Sciences, MIGAL—Galilee Research Institute, Kiryat Shmona, Israel; 4 Department of Nephrology and Hypertension, Galilee Medical Center, Nahariya, Israel; Hospital Universitario de la Princesa, SPAIN

## Abstract

Hypoalbuminemia of Hemodialysis (HD) patients is an independent cardiovascular risk factor, however, there is no mechanistic explanation between hypoalbuminemia and vascular injury. In the event of oxidative stress and inflammation to which HD patients are exposed, albumin is oxidized and undetected by common laboratory methods, rendering an apparent hypoalbuminemia. We wanted to show that these circulating modified oxidized albumin molecules cause direct vascular damage, mediating inflammation. Once these *in-vivo* albumin modifications were reduced *in- vitro*, the apparent hypoalbuminemia concomitantly with its inflammatory effects, were eliminated. Albumin modification profiles from 14 healthy controls (HC) and 14 HD patients were obtained by mass spectrometry (MS) analyses before and after reduction *in- vitro*, using redox agent 1,4 dithiothreitol (DTT). Their inflammatory effects were explored by exposing human umbilical endothelial cells (HUVEC) to all these forms of albumin. Albumin separated from hypoalbuminemic HD patients increased endothelial mRNA expression of cytokines and adhesion molecules, and augmented secretion of IL-6. This endothelial inflammatory state was almost fully reverted by exposing HUVEC to the *in-vitro* reduced HD albumin. MS profile of albumin modifications peaks was similar between HD and HC, but the intensities of the various peaks were significantly different. Abolishing the reversible oxidative modifications by DTT prevented endothelial injury and increased albumin levels. The irreversible modifications such as glycation and sulfonation show low intensities in HD albumin profiles and are nearly unobserved in HC. We showed, for the first time, a mechanistic link between hypoalbuminemia and the pro-inflammatory properties of *in-vivo* oxidized albumin, initiating vascular injury.

## Introduction

Serum albumin, the most abundant antioxidant protein in plasma, is a negative acute-phase protein associated with inflammation. In clinical states associated with chronic inflammation and oxidative stress (OS), hypoalbuminemia is prevalent [1]. Hypoalbuminemia is an established predictor of all cause and cardiovascular disease (CVD) death in hemodialysis (HD) patients [2], however, a lack of causal explanation between hypoalbuminemia and CVD morbidity and mortality, still exists.

Albumin can be found in blood as a mixture of its reduced cys-34 form (Human Mercapto-albumin, HMA) and oxidized forms (non-HMA), resulting from different states of oxidation [3,4]. Only some of these oxidative modifications are reversible [5]. We have shown that oxidative modifications of albumin impair its quantification by the standard laboratory albumin assay, bromo-cresol green (BCG) and the gold standard nephelometry, rendering part of the sera albumin undetected [6], causing apparent hypoalbuminemia in HD patients.

In addition to the disturbance of its biological functions (such as decreased antioxidant activity[7]) oxidized albumin can mediate inflammatory reactions. This was shown *in-vitro* via neutrophil activation process [6]. Serum albumin isolated from HD patients also showed similar effect on neutrophil activation ex-vivo [8]. Treatment of human umbilical vein endothelial cells (HUVEC) with *in-vitro* oxidized albumin (modified by advanced glycation end-products, AGEs), promoted mRNA expression and secretion of TNF-α as well as a reduction in expression of eNOS [9].

Cysteinylation, the major mechanism of protein–thiol modification [10] is a reversible non-enzymatic post-translational modification, abundant in patients with chronic kidney disease [11]. Upon beginning of renal replacement treatment, due to the OS induced by hemodialysis, the thiol levels are further decreased [12,13]. However these decreased levels of reduced thiol were not, as of yet, linked to the patients' hypoalbuminemic state. In addition, the inflammatory properties of *in-vivo* oxidized albumin were mainly related to its irreversible modifications such as carbonylation and glycation [6,8,9].

We imply that in HD patients the modified circulating serum albumin is associated with ongoing increase in endothelial damage and cardiovascular morbidity. Yet, the direct effects of *in-vivo* oxidized albumin and the link among specific albumin modifications, hypoalbuminemia and endothelial functions, have not been explored.

The aim of this study was to clarify the mechanistic link between hypoalbuminemic state and inflammation, possibly to suggest a new causal explanation for hypoalbuminemia as a predictor of cardiovascular morbidity. Particularly, this study aimed to determine the unique *in-vivo* albumin modifications profile of HD patients, to evaluate its pro-inflammatory effects on endothelial cells and to correlate these effects to albumin reversible and/or irreversible modifications.

## Materials and methods

### Chemicals

Acetonitrile and formic acid (HPLC supra-gradient grade) were purchased from Bio-lab Ltd. (Jerusalem, Israel), and 1, 4 dithiothreitol (DTT) was purchased from Roche (Basel, Switzerland). All other reagents were of the highest purity available and were purchased from Sigma-Aldrich (St Louis, MO, USA).

### Patients and healthy controls

The subjects enrolled in this study included 14 End Stage Renal Disease patients (4 females, 10 males, age range 40–65 years), on chronic HD therapy (4 h, thrice weekly for over a year) (see S1 File) and 14 age and gender matched healthy controls (HC), (9 males, 5 females, age range 45–70 years) (see S1 File). HD patients were diabetic and hypoalbuminemic according to the hospital clinical laboratory (Aeroset chemical analyzer, Abbott Laboratories, Abbott Park, IL, USA) the normal range is above 3.8 (serum albumin 2.5–3.7 g/dL). All patients were dialyzed using High Flux FX10 Polysulfone Dialyzers (Fresenius Medical Care), and ultra-pure water. Blood was always drawn from the arterial line before the initiation of dialysis. All showed normal liver function and no evidence of infection, malignancy, or severe hyperparathyroidism. This study took place at Galilee Medical Center in Naharia, Israel between the years 2013–2016 and was approved by our Medical Center's IRB (#62710) and by the Ministry of Health (#920100296); written informed consent was obtained from all subjects enrolled in this study. All participants who were approached, agreed to participate in this research with no drop outs.

### Isolation of serum albumin from HD patients and HC sera

Albumin was isolated from sera using Cibacron Blue 3GA Agarose (CB3GA) according to Michelis et al [14]. Following the depletion of immunoglobulin contaminations, the resin-bound immunoglobulins were discarded by centrifugation and the supernatant containing the extracted albumin, was concentrated using a 30,000 molecular-weight cut-off (MWCO) polyethersulfone column (VS0221-Vivaspin, Sartorius Stedim Biotech GmgH, Goettingen, Germany) and used for Mass Spectrometry analysis and further experiments.

### Determination of "albumin–detection index"

Albumin detection index (ADI) is defined as the ratio between the readout of the albumin-specific assay BCG to the total albumin concentration in the fraction as determined at optical density 280 nm (OD280) [15]. The BCG assay was performed according to instructions provided with the Aeroset chemical analyzer (Abbott Laboratories, Abbott Park, IL, USA). Thus, ADI = 1 when the fractionated albumin shows similar concentrations by the BCG and the OD280 assays. When the BCG-measured concentration decreases as a result of albumin modifications, the ADI will be <1 [15].

The purity of albumin was confirmed by Mass Spectrometry (MS) and SDS- gel electrophoresis.

### In-vitro albumin reduction with redox agent 1,4 dithiothreitol (DTT)

Isolated serum albumin samples (0.01 mM) from 5 HD patients and 5 HC subjects were incubated with a 1 mM solution of DTT at 37°C for 1 h. At the end of the incubation, DTT was removed using a 30,000 MWCO PES cut-off column (VS0221-Vivaspin), and the samples were analyzed using liquid chromatography/mass spectrometry quadrupole time-of-flight (LC/MS-QTOF).

### Albumin modifications analyses

Mass analyses were carried out using Agilent 6540 UHD accurate-mass Q-TOF LC/MS coupled with ultra-high performance liquid chromatography (UHPLC) 1290 (Agilent Technologies, Santa Clara, CA, USA). The instrument was set to positive ion mode, the ion source being Dual ESI (Electrospray Ionization). Gas temperature was set at 325°C, gas flow rate at 8 L/min, nebulizer gas at 40 psig, fragmentor at 280, and acquisition time was 18 min. Metabolites were separated using a Zorbax RRHD C18 column (1.8 μm, 2.1 × 50 mm, Agilent) with a mobile phase consisting of acetonitrile + 0.2% formic acid and DDW + 0.2% formic acid. The deconvoluted albumin masses and the area under each peak mass were obtained using Bioconfirm software (Agilent). Percentage of total area (% total area) under each mass peak was calculated by dividing the area under each peak mass by the total area under all considered peaks.

### Primary cultures of HUVEC

Endothelial cells were obtained from human umbilical cord veins after deliveries, upon signing of informed consent in accordance with the Helsinki declaration, approved by the IRB (#62710) and by the Ministry of Health (#920100296). Cell isolation from the cord veins and their proliferation are described elsewhere [16]. All experiments were conducted with cells after 2 to 4 passages. HUVEC were cultured in M-199 medium (Biological Industries, Israel) containing 20% fetal calf serum (FCS), 1% Penicillin-Streptomycin-Neomycin (PSN), 1% L-glutamine, 0.07% heparin and 5 μL/mL of endothelial growth mitogen (EGM, Biomedical Technologies, Thermo Fisher Scientific). When cells reached 80–90% confluency, the medium was removed, and the cells were washed twice with PBS. Unless mentioned otherwise, experiments were performed in six-well tissue culture plates (Nunc, Roskilde, Denmark) in assay medium consisting of complete medium, depleted of FCS and supplemented with 2% BIOGRO-2 serum-albumin-free (Biological Industries, Israel), for 4 h in the presence of 1 mg/mL of HC albumin or HD albumin. The procedure included a 1 h medium deprivation in assay medium before the start of the experiments. To minimize the effect of the HUVEC origin on the results, an experiment, where the effects of HD and HC albumin were assessed, was carried out using the same umbilical cord (n = 1). Nevertheless, a different cord was used for each repetitive experiment (where n>1).

### RNA isolation, reverse transcription PCR and quantitative real time PCR

Total RNA was isolated using the Quick-RNA™ Microprep Kit (Zymo Research, Irvine, CA, USA) according to the manufacturer’s instructions. RNA quality was assessed using the NanoDrop ND-1000 spectrophotometer. RNA (100–500 ng) was transcribed to generate cDNA using a high capacity cDNA reverse transcription kit (Applied Biosystems, Foster City, CA, USA) with an optimized blend of random hexamers and oligo(dT) primers, according to the manufacturer’s instructions. Quantitative real-time PCR was performed using the StepOnePlus™ system (Applied Biosystems). The mRNA levels of the inflammatory genes IL-6 (Hs.PT.58.40226675), IL-1-ß (Hs.PT.58.1518186), and IL-8 (Hs.PT.58.39926886), the enzyme eNOS (Hs.PT.58.21263066) and the adhesion molecules ICAM-1 (Hs.PT.58.4746364) and VCAM-1 (Hs.PT.58.19511717) were measured using a qPCRBIO probe mix Hi-ROX (PCR Biosystems, London, UK) according to the manufacturer’s instructions. Both human glyceraldehyde 3-phosphate dehydrogenase (GAPDH, Hs.PT.39a.22214836) and human large ribosomal protein (RPLPO, Hs.PT.39a.22214824) were used as control housekeeping genes. Melting curve analysis was performed to confirm amplification of specific transcripts. Each reaction was run in triplicate and in parallel. The expression levels of transcripts were calculated by the relative quantification (ΔΔCt) study method using SDS software (Applied Biosystems). All primers and probe sequences were purchased from IDT (Integrated DNA Technologies, Coralville, IA, USA).

### IL-6 secretion by HUVEC

HUVEC were treated for 6 h with 1 mg/mL of albumin extracted from either HC or HD patients. The conditioned medium from each well was then collected and frozen (–80°C). IL-6 levels in cell culture medium were measured by enzyme-linked immunosorbent assay (ELISA, R&D Systems, Minneapolis, MN, USA).

### Statistical analysis

Statistical evaluation of sample size: The size of n = 14 for each group (HD and controls) was calculated for a power of 80%, α = 0.05, with a standard deviation that was evaluated as 1/6 of the total range.

Differences between the two subjects' groups (HC and HD) were evaluated by using the non-parametric Mann Whitney test (two-tailed). The Wilcoxon test was applied when comparing samples before and after *in-vitro* albumin reduction with DTT. Data are expressed as mean ± S.D. All statistical analyses were performed using the GraphPad Prism software 6.00 (Graph-Pad Prism Software, Inc., San Diego, CA, USA), with statistical significance set at *P* < 0.05. All MS profiles were performed using the OriginPro 2015 software (OriginLab, Massachusetts, USA).

## Results

### Patients' albumin is highly oxidized, with thiol group cysteinylation

**Fig 1:**
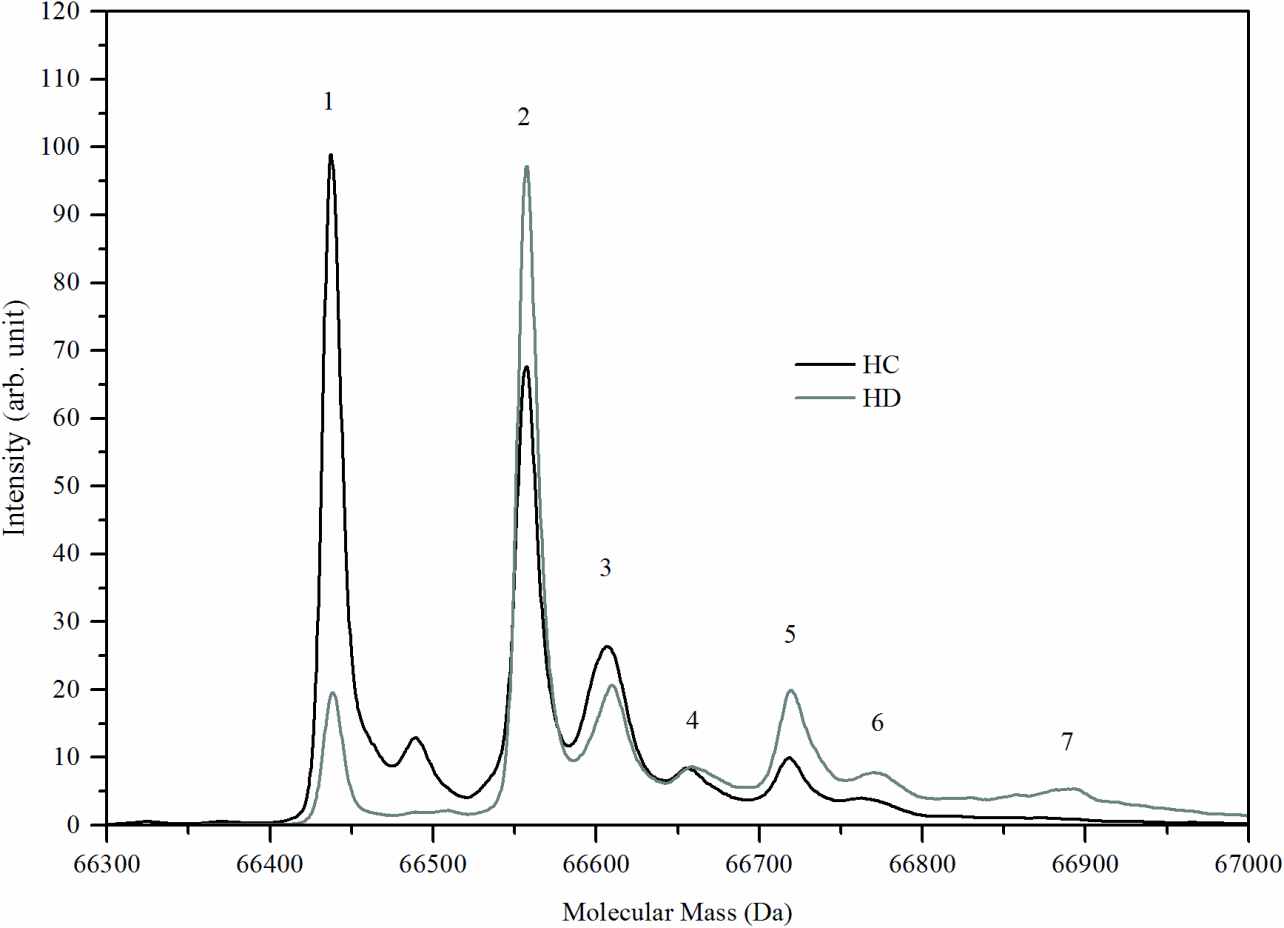
Profile of albumin modifications (n = 14). An averaged MS spectrum of isolated albumin from 14 healthy controls (HC) and 14 HD patients (HD). (see S1 File).

The MS average spectrum of HC and HD groups is shown in Fig 1. The deconvoluted masses and the different albumin compounds and their description are summarized in Table 1. Seven characteristic peaks of albumin compounds were detected and their identities were determined based on the difference between each observed mass and the reduced cys-34 form of albumin (peak 1) (Δ Da, Table 1). Albumin masses comprise the percentage of total area under each peak relative to the total area under all considered peaks. Among healthy subjects, the Human Mercapto Albumin (HMA, peak 1, Fig 1) was the most abundant and comprised 29.7±3.7 of the total area (Table 1). Conversely, among HD patients, this peak comprised only 11.9±5.3 of the total area (Table 1) while cysteinylated albumin (Cys-alb) was the most abundant form (peak 2, Fig 1). When combined, all irreversible modifications (peaks 3–7) accounted for 46.4±5.5% of the total area in HC and 59.6±6.3% in HD.

**Table 1:** Identification of albumin compounds. The identity of seven characteristic peaks of albumin compounds was determined based on the differences between each observed mass and the reduced cys-34 mass [4,17].

Peak	Average of observed mass (Da)	Δ Da	% of total mass		Compound [4,17]	Description
			HC	HD		
1**	66439.0±1.3	0.0	29.7±3.7	11.9±5.3	HMA	Human mercapto- albumin: Albumin with a free thiol at position 34
2*	66558.6±1.3	119.6	23.9±2.8	28.5±5.4	Cys-alb	Cysteinylated albumin: A cysteine bound to the free thiol at position 34 of albumin
3	66606.3±1.6	167.4	21.2±2.7	15.7±5.6	Alb + 1glyc	Albumin with a free thiol at position 34 + 1 glycation
4	66658.6±2.7	219.6	9.2±3.2	14.6±4.5	Alb+2HSO_3_	S-S reduction and the formation of 2 sulfonic acids
5	66721.8±3.0	282.8	10.8±2.4	14.3±3.5	Cys-alb + 1glyc	A cysteine bound to the free thiol at position 34 of albumin + 1 glycation
6	66764.3±4.1	325.3	6.6±2.1	9.3±4.1	Alb + 2glyc	Albumin with a free thiol at position 34 + 2 glycations
7	66880.5±2.9	441.6	2.2±1.8	5.9±2.0	Cys-alb + 2glyc	A cysteine bound to the free thiol at position 34 of albumin + 2 glycations

^*^P < 0.05 and

^**^P < 0.001, HC vs. HD

**Fig 2:**
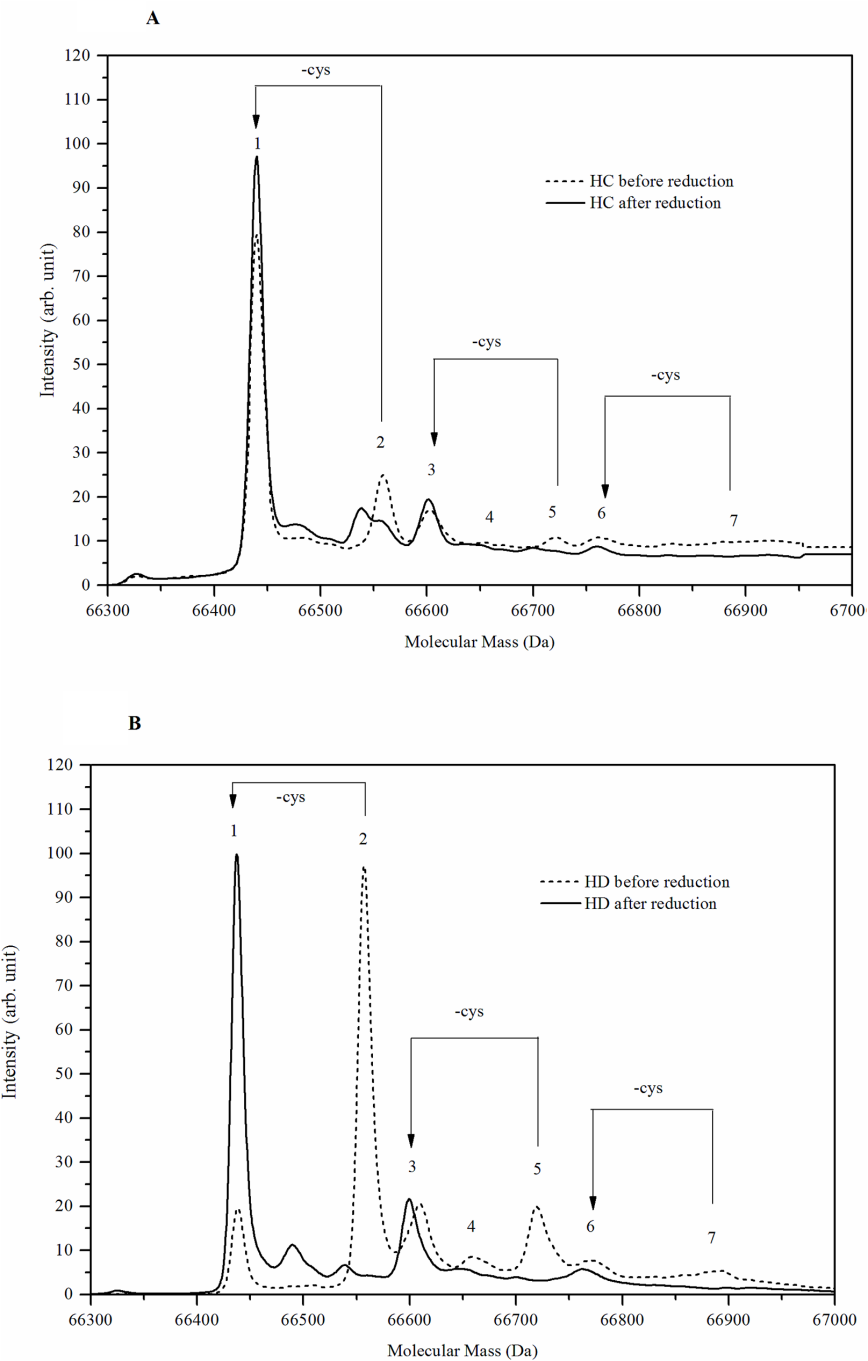
Profile of albumin modifications before and after *in-vitro* reduction. **(A)** An averaged MS spectrum of albumin isolated from 5 healthy controls (HC) before and after *in-vitro* reduction. **(B)** An averaged MS spectrum of HSA isolated from 5 hemodialysis patients (HD) before and after *in-vitro* reduction. Peak numbers are described in Table 1. (see S1 File).

In order to further evaluate and define the nature of the modifications present in HC and HD patients, five isolated albumin samples from each group were reduced by the redox agent DTT, prior to MS analysis (Fig 2A and 2B). As a result of the reduction, all cysteinylated albumin compounds, Cys-alb (peak 2), Cys-alb + 1glyc (peak 5), Cys-alb + 2glyc (peak 7) were completely removed. Simultaneously, a corresponding elevation in the area of HMA (peak 1), Alb + 1glyc (peak 3), Alb + 1glyc (peak 6) were observed (Fig 2A and 2B). The incidence of the peaks before and after the reduction is summarized in a Table 2.

**Table 2:** The incidence of albumin compounds after reduction. Results shown as mean ± SD.

	% of total area			
Peak	HC before reduction[n = 5]	HC after reduction[n = 5]	HD before reduction[n = 5]	HD after reduction[n = 5]
1^***^	37.5±5.3	52.9±4.6	20.2±1.9	51.0±2.6
2^***^	24.7±2.1	0	31.2±2.3	0
3^***^	14.2±1.0	28.3±6.3	16.4±1.4	26.9±1.4
4	9.7±1.1	5.1±6.9	9.7±1.0	11.1±2.3
5^***^	8.5±1.1	0	13.4±1.6	0
6^*^	5.3±1.4	13.7±5.2	5.5±1.3	11.0±2.6
7	0	0	3.7±1.1	0

^*^P < 0.05

^***^P < 0.01, before vs. after reduction for both: HC and HD

### HD albumin increases inflammatory response in endothelial cells

To elucidate the pro-inflammatory effects of modified albumin, HUVEC were exposed to serum albumin separated from HC subjects and HD patients. The concentration of albumin used had no effect on cell viability (data not shown), allowing the affected cells to remain attached and analyzed.

**Fig 3:**
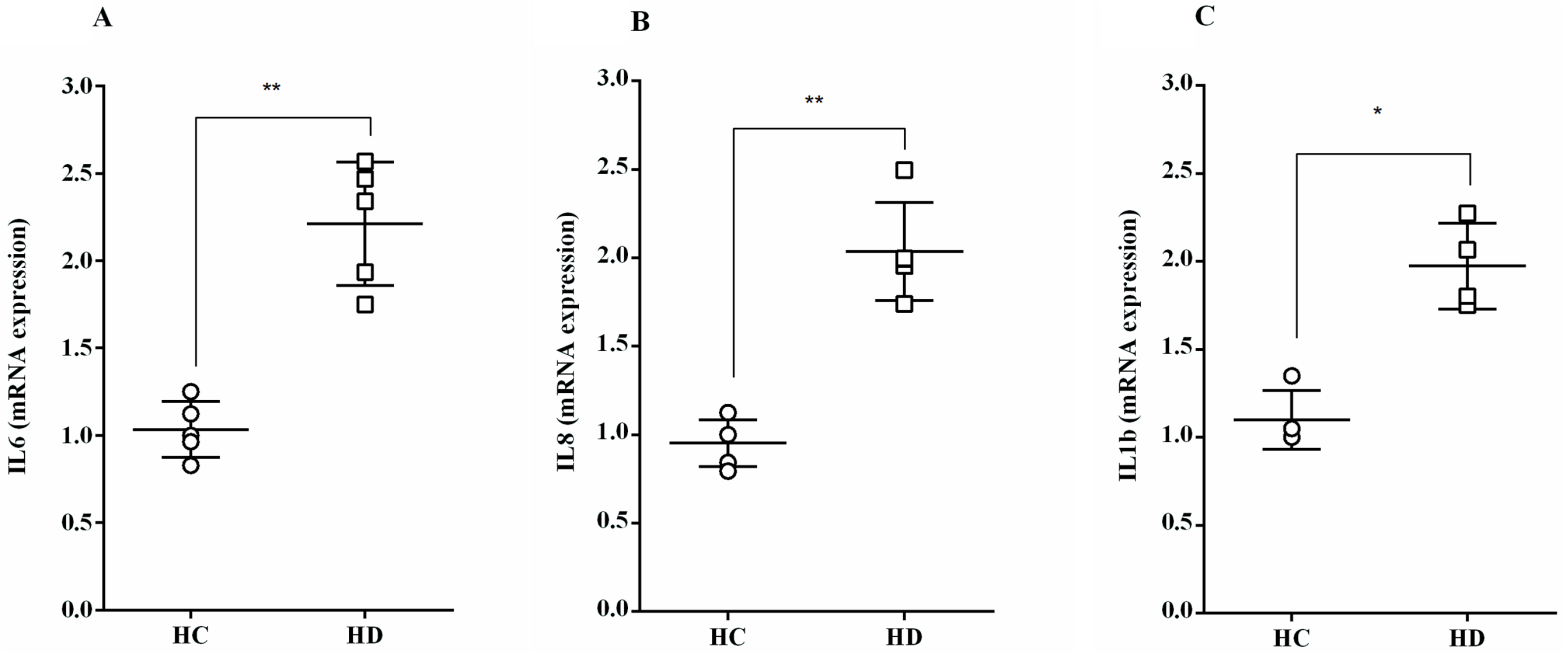
The effect of albumin from HD patients on mRNA expression of inflammatory cytokines. **(A)** Interleukin 6 (IL-6, n = 5), **(B**) Interleukin 8 (IL-8, n = 5), **(C**) Interleukin 1ß (IL-1-ß, n = 4). ^*^P < 0.05 and ^**^P < 0.01, HC vs. HD (see S1 File).

The mRNA expression of the inflammatory cytokines, IL-6, IL-8 and IL-1ß, increased significantly following the 4 h exposure of HUVEC to HD patient's albumin (2.21 ± 0.16, 2.04 ± 0.12 and 1.97 ± 0.12-folds respectively), compared to HC albumin (Fig 3).

**Fig 4:**
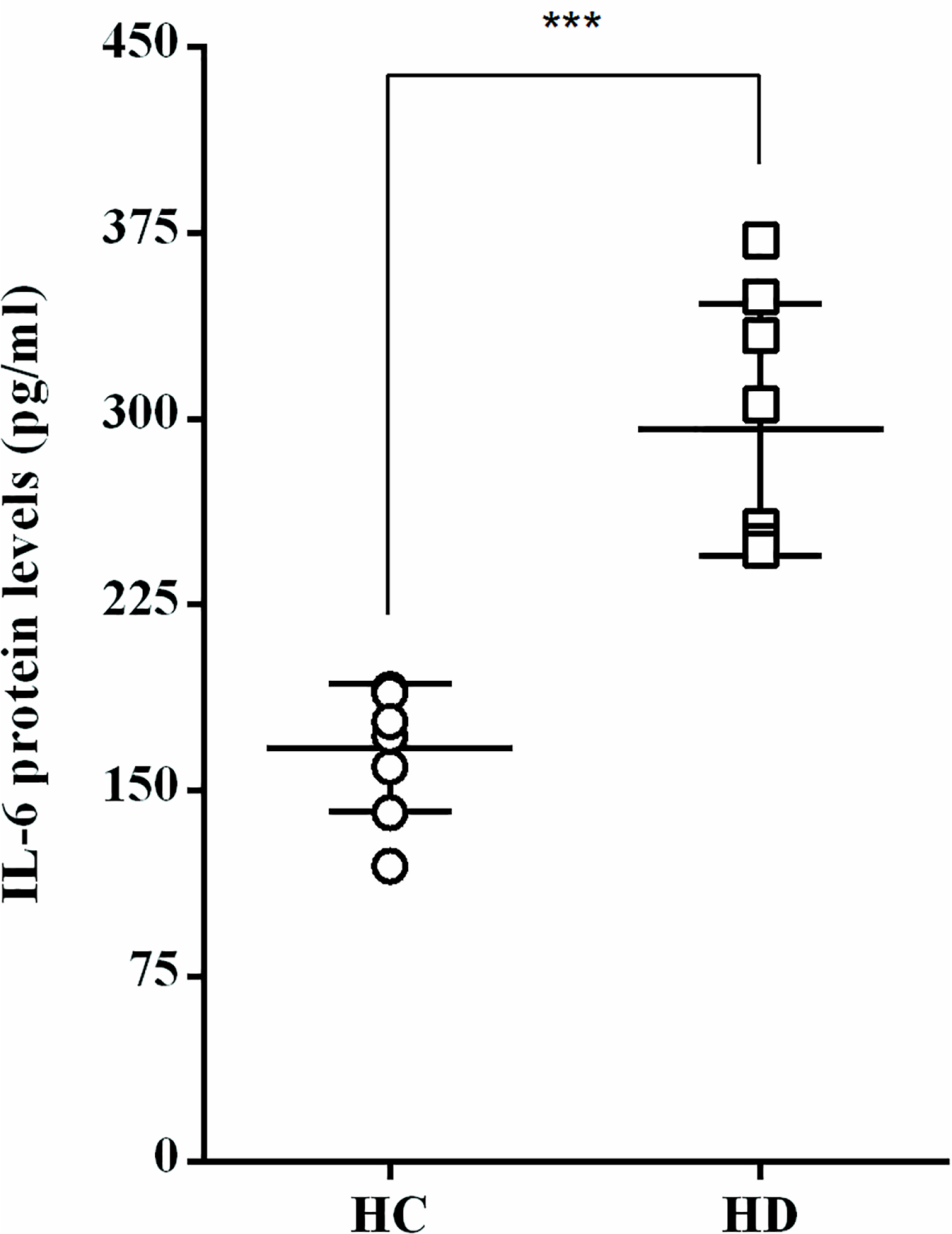
IL-6 protein levels released from HUVEC exposed to HD albumin. n = 8, ^***^P < 0.005, HC vs. HD. (see S1 File).

The protein levels of secreted IL-6 were also significantly increased in HUVEC incubated with albumin of HD compared with HC albumin (295.6 ± 51.0 and 167.3 ± 25.9 pg/mL, respectively) (Fig 4).

**Fig 5:**
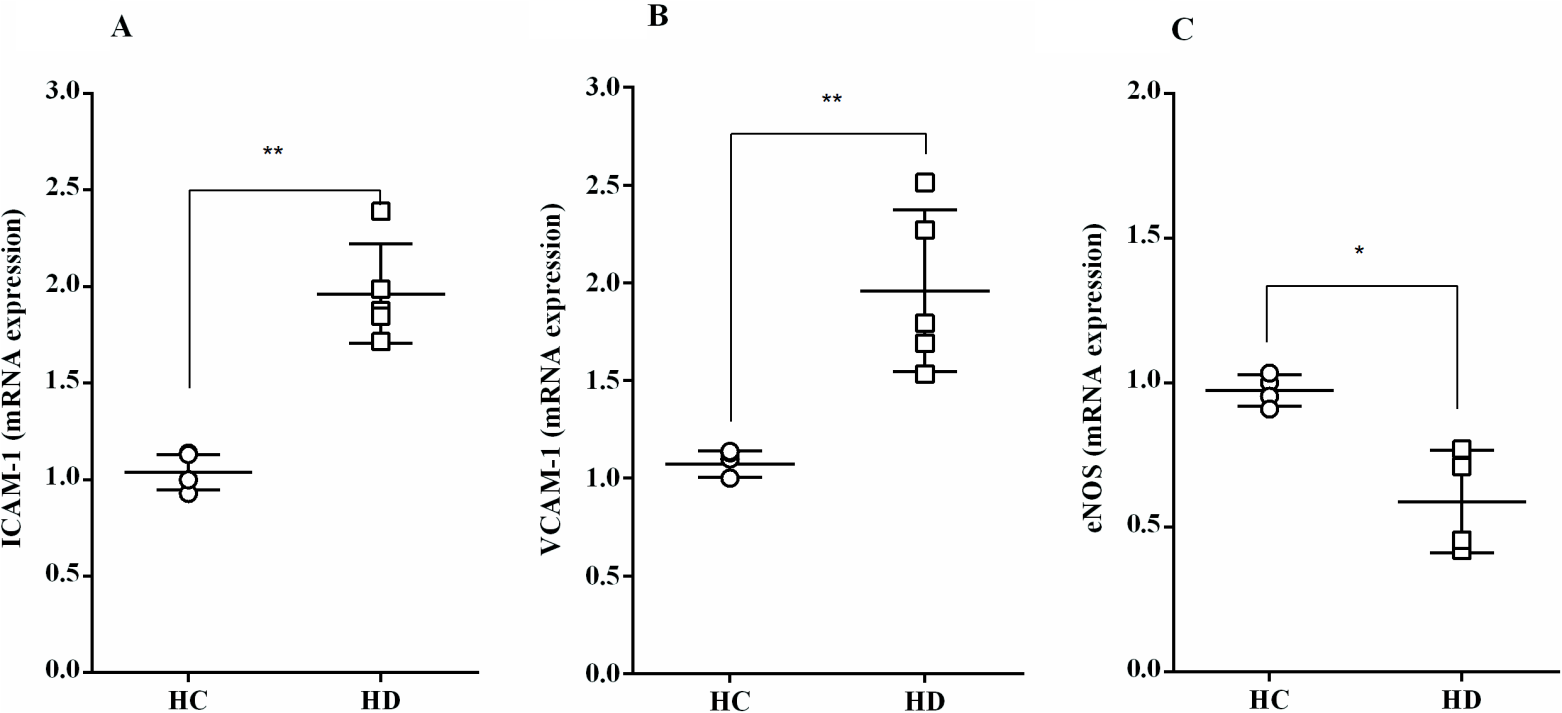
The effect of albumin from HD patients on mRNA expression of adhesion molecules and eNOS gene expression in HUVEC exposed to HD albumin. (**A**) ICAM-1 (n = 5), **(B)** VCAM-1 (n = 5), **(C)** eNOS (n = 4), ^*^P < 0.05, ^**^P < 0.01, HC vs. HD. (see S1 File).

The mRNA expression levels of the adhesion molecules, ICAM-1 and VCAM-1 following the incubation with HD albumin, increased significantly compared to HC albumin (1.57 ± 0.11 fold increase for ICAM and 2.14 ± 0.46-fold, for VCAM, Fig 5A and 5B).

Moreover, the mRNA expression of eNOS was significantly reduced after HUVEC treatment with albumin of HD patients (0.59 ± 0.09-fold, Fig 5C) compared to HC.

### The inflammatory effect of HD-albumin is mainly due to reversible modifications in albumin

**Fig 6:**
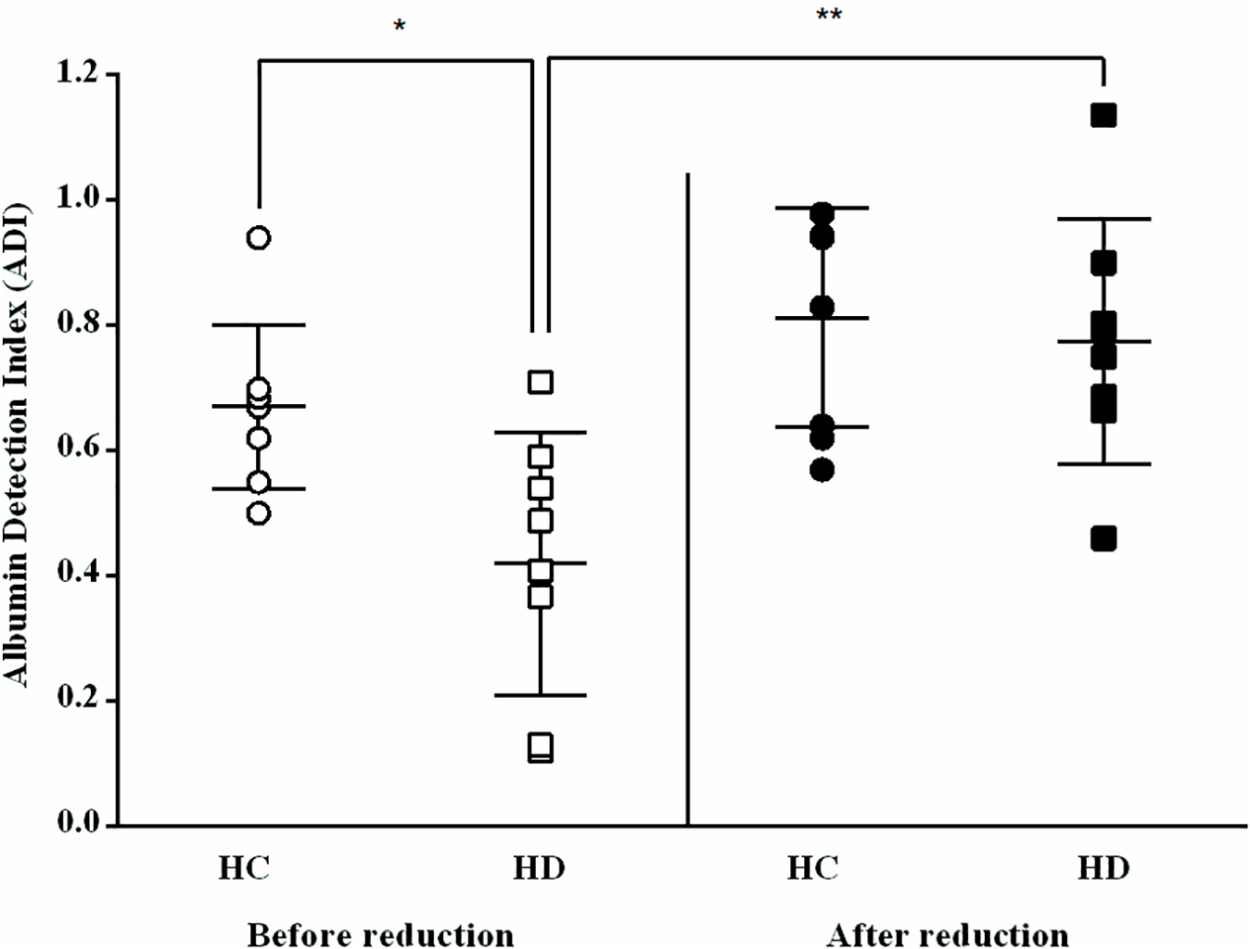
The "albumin detection index -ADI" after *in-vitro* reduction of albumin. ^**^P < 0.01, ^*^P < 0.05, HC vs. HD before reduction vs. after reduction (n = 8). (see S1 File).

After reduction *in-vitro* of the separated sera albumin from HC and HD patients, the calculated ADI increased significantly in HD patients, reaching HC-comparable levels (Fig 6), while the HC ADI, which were primarily significantly higher than HD, remained unchanged.

**Fig 7:**
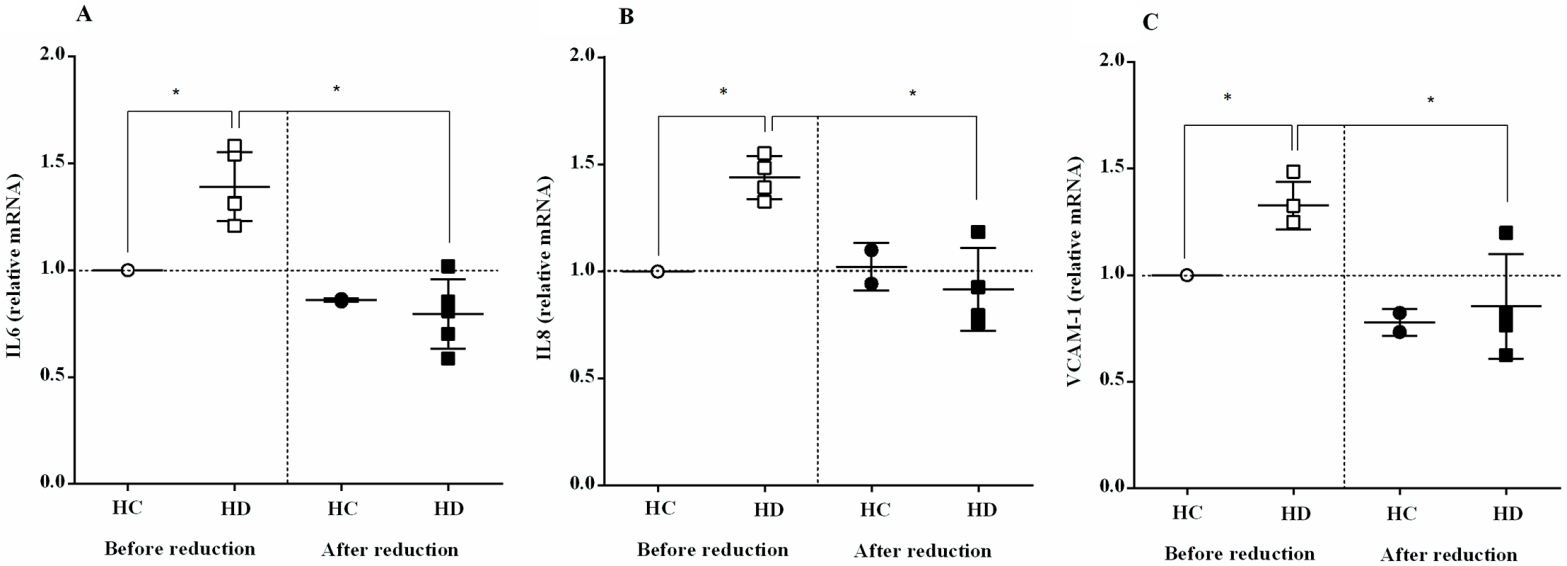
*In-vitro* reduced albumin from HD patients decrease the mRNA expression of inflammatory cytokines and adhesion molecules. **(A)** IL-6 (n = 5), **(B)** IL-8 (n = 4), **(C)** VCAM-1 (n = 5). ^*^P < 0.05, before reduction vs. after reduction. (see S1 File).

The mRNA expressions of IL-6 and IL-8 and of VCAM-1 were determined in HUVEC before and after albumin reduction with DTT (Fig 7A–7C). The mRNA expression levels of these inflammatory cytokines and the adhesion molecule decreased after reduction of HD albumin, reaching levels equal to or even below the reference RQ = 1 (all results were normalized to the expression of these genes after exposure to HC albumin), indicating that *in-vitro* reduced HD albumin does not promote endothelial inflammation. In addition, a Pearson product-moment correlation coefficient was computed to assess the relationship between mRNA expressions (IL-6, IL-8 and VCAM) and ADI, showing significant negative correlations between them: IL-6 (r = -0.674, n = 10, p = 0.033), IL-8 (r = -0.675, n = 10, p = 0.032) and VCAM-1 (r = -0.697, n = 12, p = 0.012). Overall, a decrease in mRNA expressions correlated with an increase in ADI.

## Discussion

We show herein that *in-vivo* modified albumin, separated from hypoalbuminemic HD patients is a pro-inflammatory mediator of endothelial cells. This endothelial inflammatory state was almost fully reverted by exposing HUVEC to the *in-vitro* reduced HD albumin.

An albumin modifications profile was characterized for HD patients and for healthy subjects (HC). The profile of albumin modifications peaks was similar between HD and HC, but the intensities of the various modifications were significantly different: HMA (peak 1) and the reversible cysteinylation modification (peak 2), present in HC and HD, are interchangeable peaks, as they can be easily manipulated by *in-vitro* reduction. All other modified albumin forms that represent irreversible modifications such as glycation and sulfonation (peaks 3–7), show low intensities in HD albumin profiles and are nearly unnoticed in HC.

Recent studies using mass spectrometry demonstrated that albumin cysteinylation in chronic diseases [4,11], and in sera of end stage renal disease patients in particular [13], is greater than in healthy subjects. As a result of the cysteinylation, some of the beneficial activities attributed to serum albumin, especially its antioxidant activity, are hampered [11,18].

After the *in-vitro* reduction of HD albumin, an increase in the percentage of the reduced form and a decrease in the cysteinylated form of albumin were found, concomitantly with significant improvement in the pro-inflammatory properties of endothelial cells being exposed to the *in-vitro* reduced albumin. It should be noted that the *in-vitro* reduced HD albumin sample, showed a significant increase in albumin concentration followed by an increase in ADI.

In the present study, albumin separated from HD sera, with low albumin index due to oxidative modifications, triggered an inflammatory response in endothelial cells, increasing mRNA expression of the inflammatory cytokines, IL-6, IL-8 and IL-1β, as well as higher levels of IL-6 protein secretion. We show that oxidized HD albumin upregulated the adhesion molecule VCAM in endothelial cells, an effect that was significantly reversed by *in-vitro* reduction of the HD albumin. This is supported by previous reports showing that under inflammatory conditions, levels of cell adhesion molecules such as ICAM-1 and VCAM-1, are up-regulated in order to promote monocyte infiltration via activated endothelium [19]. In addition, in chronic kidney disease, serum levels of inflammatory markers, such as IL-6, IL-8, IL-1β, are increased [20–22]. A clear link between sera inflammatory mediators such as CRP, TNF-α, IL-6 and oxidative markers such as AOPP [6,15] and albumin levels [14] were already shown, with a specific association between the levels of oxidized albumin reflected by ADI and the markers of systemic inflammation [14]. The significant negative correlation between the expression of endothelial function markers and the albumin detection indices presented herein, supports this notion: higher levels of oxidized albumin correlates with higher expression of inflammatory cytokines (IL-6, IL-8) and adhesion molecules (VCAM).

We show that treatment with HD albumin decrease endothelial NO synthase (eNOS) expression in endothelial cells compared to HC albumin. This decrease in mRNA can lead to lower NO levels, playing a role in endothelial dysfunction prevalent in these patients [23]. This predominant NOS isoform, eNOS, in the vasculature, is responsible for most of the NO produced in healthy vasculature [24].

In our study, utilizing the endogenously (*in-vivo*) oxidized albumin of HD, we show that when the reversible modifications were eliminated, endothelial injury was prevented: The ex-vivo reduction obliterated the pro-inflammatory properties of HD albumin.

Some pro-inflammatory effects of oxidized albumin, though oxidized *in-vitro*, were shown by Rashid et al. [9], where AGE-albumin, an irreversible modification, promoted mRNA expression and secretion of TNF-α, and a reduction of eNOS mRNA and protein expression in HUVEC. To the best of our knowledge, our study is the first report where endogenously oxidized albumin was used to induce endothelial activation and inflammation.

We acknowledge that the number of albumin samples used to investigate the effects of HD albumin on inflammation was limited due to the complexity of methods, however the results were consistent for various markers emphasizing the overall inflammatory effects of oxidized albumin.

We imply that hypoalbuminemia is a predictor of cardiovascular morbidity and mortality due to vasculopathy mediated by oxidized modified undetected albumin.

This implication also serves beyond HD patients, e.g. CKD patients, where albumin detection index is low [15], and indicative of the existence of a mixture of HMA and oxidized albumin molecules (non-HMA).

The albumin modifications profile enables us to relate the pro-inflammatory effects to specific types of modifications, i.e. reversible vs. irreversible. In summary, our results show that oxidized albumin from HD patients may actually be a perpetrator of endothelial activation, as demonstrated by its effects on inflammatory endothelial markers, both at the transcriptional and post translational levels, in addition to the well-known loss of its beneficial activity as an antioxidant [25].

Since the injurious albumin modifications in HD are the reversible ones, we suggest that the identification of oxidized albumin is a possible marker for use in the clinic. Since the redox status of the free thiol group in proteins can serve as an important indicator of oxidative stress [11,18], and as shown here, modification of Cys-34 in serum albumin may reflect the degree of oxidation in HD patients and serve as a simple diagnostic biomarker. This biomarker can be further utilized as a therapeutic target in decelerating the development of cardiovascular disease.

## Supporting information

S1 File Supporting information. (XLSX)
